# Gene spatial integration: enhancing spatial transcriptomics analysis via deep learning and batch effect mitigation

**DOI:** 10.1093/bioinformatics/btaf350

**Published:** 2025-06-13

**Authors:** Rian Pratama, Jason Hilton, J Michael Cherry, Giltae Song

**Affiliations:** School of Computer Science and Engineering, Pusan National University, Busan 46241, South Korea; Department of Genetics, School of Medicine, Stanford University, Stanford, CA 94304, United States; Department of Genetics, School of Medicine, Stanford University, Stanford, CA 94304, United States; School of Computer Science and Engineering, Pusan National University, Busan 46241, South Korea; Center for Artificial Intelligence Research, Pusan National University, Busan 46241, South Korea

## Abstract

**Motivation:**

Spatial transcriptomics (ST) is a groundbreaking technique for studying the correlation between cellular organization within a tissue and its physiological and pathological properties. Every facet of spatial information, including cell/spot proximity, distribution, and dimensionality, is significant. Most methods lean heavily on proximity for ST analysis, each resulting in useful insights but still leaving other aspects untapped. In addition, samples procured at different times, by different donors, and by different technologies introduce a batch effects problem that hinders the statistical approach employed by most analysis tools. Addressing these challenges, we have developed a deep learning method for analyzing integrated multiple ST data, focusing on the distribution aspect. Furthermore, our method aims to leverage single-cell analysis tools.

**Results:**

Our study introduces Gene Spatial Integration (GSI), a data integration pipeline utilizing a representation learning approach to extract the spatial distribution of genes into the same feature space as gene expression features. We employ an autoencoder network to extract spatial embedding, facilitating the projection of spatial features into gene expression feature space. Our approach allows for seamless integration of multiple samples with minimum detriment, increasing the performance of the ST data analysis tool. We show the application of our method on the human dorsolateral prefrontal cortex dataset. Our method consistently improves the performance of the clustering of Seurat tools, with the most significant increase observed in sample 151673, almost doubling the ARI score from 0.225 to 0.405. We also combine our pipeline with the clustering of GraphST, achieving a significantly higher ARI score in sample 151672 from 0.614 to 0.795. This result reveals the potential of gene distribution spatial aspect, also emphasizes the impact of integration and batch effect removal in developing a refined analysis in understanding tissue characteristics.

**Availability and implementation:**

Implementation of GSI is accessible at https://github.com/Riandanis/Spatial_Integration_GSI.

## 1 Introduction

Transcriptomics technologies have witnessed a series of innovations in recent years. One significant advancement is the introduction of single-cell omics technologies, which offer insight free of the confounding effects of genotypic or phenotypic heterogeneity present in bulk sample methods, their predecessors (Macaulay *et al.* 2017, Mincarelli *et al.* 2018). Shortly after, spatial transcriptomics (ST) technologies emerged, augmenting omics data with spatial information from cells. This advancement enables the dissection of spatial organization and intercellular interactions, aspects previously lost during the high-throughput omics data acquisition process (Aldridge and Teichmann 2020, Anderson *et al.* 2022, Moses and Pachter 2022). With ST giving us more information about cell organization, allowing us to gain a deeper understanding of gene expression patterns within their anatomical context, its application develops our understanding of certain cell impacts in a spectrum of diseases such as cancer and even Alzheimer’s (Janiszewska 2020, Ji *et al.* 2020, Rao *et al.* 2021, Chen *et al.* 2022b). The development of ST has not been slowing down yet, as technologies such as Nanostring GeoMX DSP (Van and Blank 2019) and 10× Genomics Visium (Ji *et al.* 2020, Rao *et al.* 2020) empower researchers with improved access to ST data, providing a unique point of view for distinguishing the spatial distribution of gene activity within tissues. Information that is essential to understanding the complex interactions between cells and tissues, combined with easier access to data acquisition, leads to an unprecedented trend for ST studies (Ståhl *et al.* 2016, Ji *et al.* 2020, Maynard *et al.* 2021, Chen *et al.* 2022a, Moses and Pachter 2022, Williams *et al.* 2022, Fang *et al.* 2023).

There are two categories of experimental ST techniques (Tian *et al.* 2023). First, image-based ST techniques by *in situ* hybridization and fluorescence microscopy, such as seqFISH (Shah *et al.* 2016), osmFISH (Codeluppi *et al.* 2018), and MERFISH (Zhang *et al.* 2021). Second, sequencing-based ST techniques by *in situ* next-generation high-resolution sequencing such as 10× Genomics Visium (Ji *et al.* 2020), Slide-seq (Rodriques *et al.* 2019), DBiT-seq (Liu *et al.* 2020), PIXEL-seq (Fu *et al.* 2022), Seq-Scope (Cho *et al.* 2021), and Stereo-seq (Chen *et al.* 2022a). This emerging trend is driving a new wave of capabilities that allow researchers to unveil gene expression patterns with unparalleled spatial precision. Despite this promising array of Spatial Transcriptomics techniques, our primary focus is on *in situ* sequencing-based 10× Genomics Visium Technology. This choice is driven by its current popularity, accessibility, and the wealth of information it already contains.

The expansion of ST techniques also results in a surge of spatially resolved transcriptomics data development from various sources and technologies (Moses and Pachter 2022). However, these variations in technology and protocols hinder the compatibility and integration of the data, thus impeding the establishment of cohesive spatially resolved tissue atlas analysis. Single-cell data, as the precursor to ST data, also suffers from problems related to batch effects and data variability (Cheung *et al.* 2021, Vanderaa and Gatto 2021). A common strategy to mitigate these issues is through analysis in a shared embedding space, where neural networks decode biological signals while reducing the influence of noise and batch-specific artifacts (Li *et al.* 2024). Similar to the challenges encountered in single-cell RNA sequencing (scRNA-seq), mitigating batch effects in integrated ST datasets poses a substantial hurdle. This challenge is further compounded by the additional complexities of spatial data acquisition compared to scRNA-seq, resulting in distinct batch effect characteristics (Tran *et al.* 2020, Fu *et al.* 2016).

Advances in ST analysis have incorporated solutions to the problem of batch effects within their workflows (Dong and Zhang 2022, Long *et al.* 2023, Xu *et al.* 2024), considering the spatial features of ST data during the removal of batch effects. Yet, spatial features are a complex and varied aspect of ST. Specifically, no current method uses spatial information in gene distribution to explicitly address this issue in tandem with gene expression features throughout the batch effect removal process.

Single-cell analysis tools have been essential to understand the diversity of cell types in a tissue (Aldridge and Teichmann 2020). Established tools such as Seurat (Satija *et al.* 2015) excel in various analyses, but challenges are encountered in spatial transcriptomics data analysis. The well-established nature of single-cell analysis tools is crucial for ST data analysis, as biological assays remain central in transcriptomics analysis (Tian *et al.* 2023). Optimizing single-cell analysis tools by incorporating spatial information features for ST data analysis can provide a more comprehensive understanding of tissue biology (Rao *et al.* 2021). Familiarity with single-cell analysis tools leads to the development of new directions for ST data analysis, enabling tasks such as cell type clustering, gene expression pattern mapping, and biomarker identification, which are already available in single-cell analysis tools, to be performed without significant compromise.

Significant progress has been made in the development of analysis tools for ST data. Machine learning methods, in particular, offer a leap in ST analysis, with numerous machine learning models introduced to extract information from ST data (Moses and Pachter 2022). STLearn (Pham *et al.* 2023) offers a deep learning approach to extracting tissue images contained in ST data as additional features. SpaGCN (Hu *et al.* 2021) constructs a graph network based on the nearest spatial distance among target spots, employing a graph convolutional network to learn its spatial features. STAGATE (Dong and Zhang 2022) expands the similarly constructed proximity graph network with a cell-type aware attention module for better local resolution, with the focus of separating the spatial domain between each cell. GraphST (Long *et al.* 2023) adds another spatial aspect of similarity to its graph construction process, taking into account the features of the gene expression representation of neighboring spots for edge connection. SEDR (Fu *et al.* 2016, Xu *et al.* 2024) was the first to offer an analysis on an integrated spatial transcriptomic dataset, employing a graph autoencoder in the proximity graph network to represent spatial features while also minimizing the resulting batch effects of multiple samples integration in its feature representation.

This development of analysis tools tailored for ST is currently representing a major focus of the ongoing trend for ST studies. These tools enable the extraction of deeper biological insights from ST data, facilitating new discoveries about disease mechanisms (Hunter *et al.* 2021, Liao *et al.* 2021). The development of these new methods can treat ST tissue slices as hyperspectral images (Rasti *et al.* 2020), focusing on spatial proximity and local features but leaving much of the spatial distribution and patterns insights untapped (Imani and Ghassemian 2020). Identifying the gap in processing spatial information into gene expression feature space and in response to trends, we introduce Gene Spatial Integration (GSI), a pipeline that combines gene expression and the distilled spatial distribution information from ST data into gene spatial distribution embeddings using representation learning, applicable in multiple integrated samples. Our approach emphasizes extracting global spatial distribution information for each gene, rather than focusing on local cell/spot proximity features, aiming to address batch effects while preserving compatibility with single-cell analysis tools. This extends the versatility and enables a more comprehensive understanding of the ST data.

## 2 Materials and methods

### 2.1 Overview of GSI

**Figure 1.**
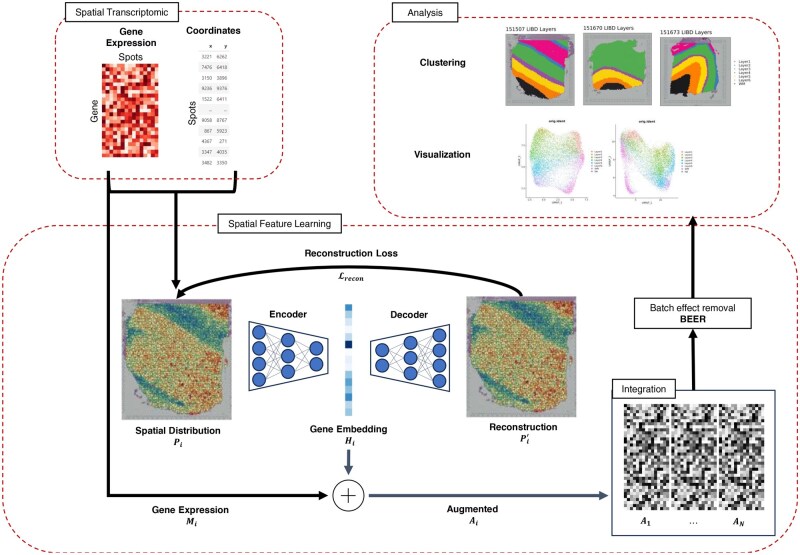
Schematic diagram of the workflow of GSI in analyzing integrated ST data. First, we collect individual samples of spatial transcriptomics (ST), each containing a gene expression assay matrix, a cell/spot coordinate list, and a tissue slice image. Next, we extract spatial features by constructing a 2D spatial distribution using count value and coordinate for each gene, utilizing an autoencoder to extract hidden embedding features from the spatial distribution images, and integrating these embedding features with the gene expression features into a single feature space. Finally, we subject our integrated dataset to a batch effect removal process before employing analysis tools to explore the insights within the integrated ST data.

The following provides an overview of our method, GSI, which employs a deep learning-based feature extraction approach, applying image-based representation learning to spatial transcriptomics data. As shown in Fig. 1, the central idea is to leverage autoencoder neural networks, a well-established method for feature extraction in 2D datasets, to capture underlying spatial information from gene distribution patterns and enhance the analysis of gene expressions within their native spatial contexts. GSI learns spatial embeddings by extracting spatial distribution and pattern features from the spatial coordinates of each gene in individual samples. By focusing on gene distribution information contained within spatial transcriptomics, we aim to explore a new dimension of spatial data beyond cell/spot proximity, which has been the primary focus of existing methods. The spatial embedding representation is then projected into the same feature space as the gene expression features, and both are combined to form augmented features. This augmented feature matrix integrates gene expression data with spatial embedding information. The resulting augmented features can be applied to various downstream analysis tasks.

Our study utilizes multiple tissue slice spatial transcriptomics samples. Each sample was processed into augmented features that represent combined gene expression and spatial embedding features before being integrated into a cohesive dataset. This integrated dataset contains important features of both gene expression and the spatial distribution of all combined samples.

This integration process presents challenges, which commonly arise when combining datasets, such as batch effects, that are also amplified because of the added spatial features. To address this, our pipeline includes a batch effect removal step to minimize its impact on the analysis. The final output is a unified dataset that integrates gene expression and spatial information from multiple samples, with batch effects reduced. The dataset is then ready for downstream analysis using single-cell analysis tools.

### 2.2 Preprocessing of spatial transcriptomics data

Our method takes information from the spatial transcriptomics dataset, in which gene expression information and spatial coordinates are contained from multiple samples. Each sample contains a count matrix $M\in R^{N_{\mathrm{gene}}\times N_{\mathrm{spot}}}$, where each cell tells a certain gene expression count at a specific spot within the sample. Gene expression counts were normalized using the Seurat pipeline, and the normalized count matrix *M* was then used as input for our method alongside the list of $N_{\mathrm{spot}}$ spot coordinates.

### 2.3 Transformation of gene spatial distribution information

To capture the spatial information of the gene distribution, we used a process of transforming the spatial coordinates of the spots of each gene in the dataset. Initially, the coordinate list was converted into a 2D numPy array, which was then treated as an image matrix *P*. This matrix represents $P\in Z^{x\times y}$, where each pixel in coordinates (*x*, *y*) denotes the presence of a gene on the plane. Subsequently, this 2D image matrix was resized to an array with dimensions of (*x* = 220, *y* = 280). During this resizing step, we utilized the nearest-neighbor algorithm to effectively handle overlapping cell pixels. Elements of *P* were defined as follows:

(1) $$p_{xy}=\{\begin{array}{cc} 1 & \text{if}\;m_{ij}>0\\ 0 & \text{otherwise} \end{array}$$

where $p_{xy}\in P$ with *x*, *y* being the coordinates of spot *j* and $m_{ij}\in M$ being the normalized count value of gene *i* on spot *j*.

This approach represents gene distribution and emphasizes the spatial information inherent in spatial transcriptomics. By analyzing gene distribution, we aim to gain a comprehensive understanding of how the spatial arrangement of genes influences the characteristics of cells within a tissue sample. Using spatial transcriptomics data as a map-like image, we investigated the correlation between gene distribution and tissue cluster formation.

### 2.4 Autoencoder-based spatial embedding extraction

We use an autoencoder to perform representation learning on gene distribution data. This approach was chosen due to the autoencoder’s ability to be trained in an unsupervised manner and its flexibility for future expansion (Meng *et al.* 2017). Autoencoders are widely used for data reconstruction, making them a go-to method for unsupervised feature extraction (Zhang *et al.* 2020). They are particularly effective in extracting spatial features from 2D maps (Egilmez and Ortega 2014, Imani and Ghassemian 2020, Cao *et al.* 2021). Since we represent spatial information in spatial transcriptomics as map-like images, the autoencoder is an ideal fit for our approach.

In our pipeline, the autoencoder took the transformed gene distribution images *P* as input, learning to reconstruct $P^{\prime }$ to represent the spatial transcriptomic image data. The autoencoder consists of two parts: an encoder and a decoder. The encoder takes the image *P* as input and transforms it into a latent representation *H*. The decoder then takes the latent representation *H* as input and transforms it back into the image $P^{\prime }$.

(2) $$L_{\mathrm{recon}}=\frac{1}{N_{\mathrm{gene}}}\sum_{i=1}^{N_{\mathrm{gene}}}\left|\right|(P_{i},P^{\prime }{}_{i})|{|}_{2}$$

By minimizing the mean square error loss function $L_{\mathrm{recon}}$, the autoencoder network is trained to adjust the parameters *W*, $b_{P_{i}}$, and $b_{H_{i}}$ to learn a representation *H* that can reconstruct the image $P^{\prime }$ as closely as possible to *P*. The hidden feature *H* is a reduced representation of the spatial information from the image *P*, which proves useful for various analysis tasks, including cell clustering, cell type identification, and gene differential expression analysis.

Our experiment implements the Autoencoder network in PyTorch (Imambi *et al.* 2021). Details of implementation are provided in Notes 1.1, available as supplementary data at *Bioinformatics* online.

### 2.5 Data augmentation: spatial embeddings and gene expression

To enhance the comprehensibility and analytical capacity of ST data, we devised a data augmentation technique that combines spatial embeddings with gene expression information into the gene expression feature space. This was achieved by concatenating the spatial embedding output from the encoder with the gene expression matrix, resulting in an augmented feature matrix containing both gene and spatial information.

(3) $$\begin{array}{c} A=\left[\begin{array}{c} M\\ H \end{array}\right] \end{array}$$

where *A* is the augmented feature matrix, *M* the gene expression features, and *H* the latent features from the encoder.

### 2.6 Sample integration with batch effect removal

The integration phase combines multiple augmented datasets from individual samples into a single set, denoted as *C*, to enhance statistical power by increasing the sample size for analysis.

(4) $$C=\left[\begin{array}{cccc} A_{1} & A_{2} & \ldots & A_{N} \end{array}\right].$$

where *C* is a matrix of integrated features consisting of *N* samples of an augmented feature matrix $A_{i}$ for *i* from 1 to *N*. Each matrix $A_{i}$ contains enhanced gene embeddings that combine gene expression information (*M*) and spatial information (*H*).

The resulting matrix *C* can then be stored in the Seurat object format and treated as assay data, which retains compatibility with the Seurat tools for seamless processing, including batch effect removal and downstream analysis.

To mitigate batch effects, we applied the Batch EffEct Remover (BEER) pipeline, which is designed to reduce batch effects in integrated scRNA-seq datasets (Zhang *et al.* 2019). We selected the BEER pipeline because its workflow aligns with the output of the previous step, spatial feature learning. It is well-suited to operate on the resulting feature space of our combined dataset, while also offering transparency in its implementation and flexibility through adjustable hyperparameters.

In our workflow, the combined feature matrix *C* is used directly as input to BEER without the need to re-encode through the feature learning network. BEER outputs a corrected version of *C*, with reduced batch-specific variations, ready for further analysis.

In addition, the combined dataset is optimized for the use of the BEER tool, which takes advantage of the correlation of the mutual nearest cell pairs identified from different batches to identify Principal Component Analysis (PCA) subspaces with poor correlation (indicates a high batch effect) while giving the option to apply the empirical Bayesian correction method (Johnson *et al.* 2007) for further correction in its process pipeline. With the BEER tool, both the gene expression information and the spatial information in matrix *C* are considered in the batch removal process.

Batch effect removal was performed using the BEER package in R, with the hyperparameters of: GNUM = 50, PCNUM = 30, ROUND = 2, GN = 1500, SEED = 1, and COMBAT = TRUE. The integrated dataset after correction, denoted $C^{\prime }$, represents the final output of our treatment with mitigated batch effects, ready for analysis.

### 2.7 Downstream analyses using Seurat

We used Seurat tools in R for analysis tasks, including clustering and differential expression analysis, using our pipeline output $C^{\prime }$ as input data. For clustering, we used the Louvain algorithm available in Seurat, tuning the resolution parameter through a grid search to identify clusters that best match the ground truth.

We find that Seurat remains the better option for transcriptomics data analysis tools. Considering the utilization of Seurat tools, our pipeline adapts its processed features to the feature space that gives high compatibility with Seurat functions.

### 2.8 Performance evaluation metrics

The evaluation metrics to measure pipeline performance in data integration were performed using biological metrics proposed by Luecken *et al.* (2022). These metrics are grouped into two categories: (i) batch mixing measurement and (ii) conservation of biological signal measurement. The batch mixing measure includes batch ASW, graph connectivity, iLISI, and kBET. The second category, conservation of biological signal, includes ARI, NMI, ASW, cell ASW, cLISI, isolated F1, and isolated ASW.

Evaluation was implemented in a Python environment using the scib library package (https://github.com/theislab/scib), using the embedding output of the measured pipeline as input. More details on the evaluation metrics are provided in Note 1.2, available as supplementary data at *Bioinformatics* online.

## 3 Results

### 3.1 Data description

We used datasets from the LIBD human dorsolateral prefrontal cortex (DLPFC) acquired with 10× Visium, as documented in Maynard *et al.* (2021), as the first dataset. These datasets are available in spatial experiment format through the SpatialLIBD library in R, as described in Pardo *et al.* (2022). We group a selection of samples based on tissue subjects to enable targeted learning by our neural network. The first group, as the benchmark set, consists of all three available donor subjects Br5292, Br5595, and Br8100, with samples 151507, 151672, and 151673 selected, as these samples were previously examined in relevant studies that address spatial transcriptomics data integration (Fu *et al.* 2016). For the second and third groups, we curate a selection consisting of four samples that belong to a single donor subject to investigate the improvements that occur in the relevant samples. This grouping is summarized in Table 1 available as supplementary data at *Bioinformatics* online.

We use spot filtering to remove spots with zero values, with the resulting number of spots in each sample ranging from 3639 to 4226, with a total of 33 538 genes captured across all samples. Additionally, each sample provided manually annotated regions consisting of 4–6 DLPFC layers and a white matter region.

The second dataset that we use is the combination of 10× Visium Mouse Brain Coronal Section 1 and Mouse Brain Coronal Section 2. Both of these datasets are available on the 10× Visium website. The Coronal Section 1 dataset contains 2310 spots, and the Coronal Section 2 dataset contains 2235 spots, both of which consist of the same 19 465 genes.

Then, we preprocess the data similar to the DLPFC dataset using the default sequence workflow of normalization and data scaling in Seurat. Then both of these preprocessed datasets are combined into an integrated dataset of 4545 spots with 19 465 genes, which we label as the Coronal dataset. However, these dataset samples do not have annotated regions that could act as ground truth domains for evaluation, unlike the DLPFC dataset.

### 3.2 GSI on sample integration and batch effect mitigation

**Figure 2.**
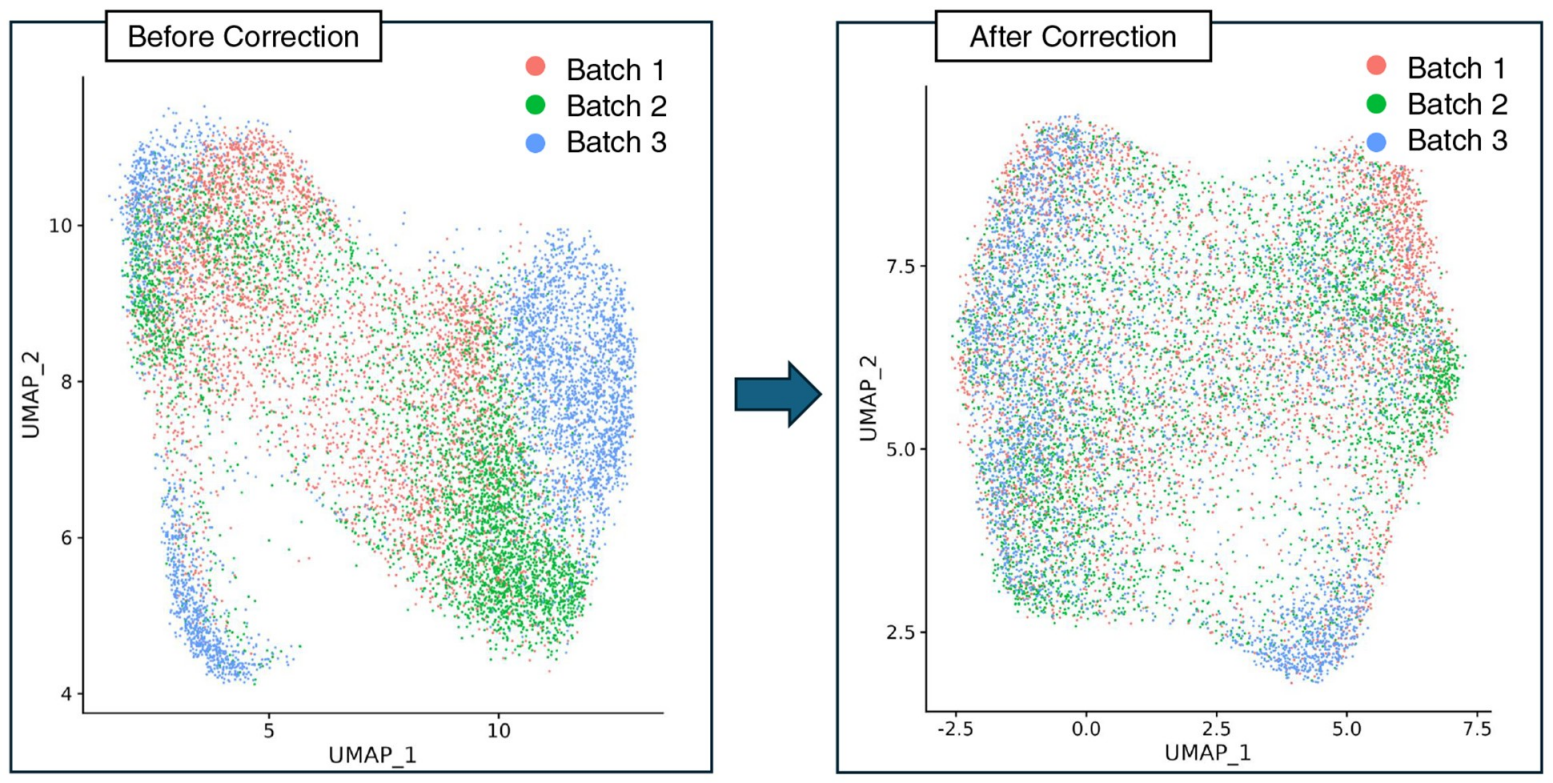
Visualization of batch clusters before and after the batch effect removal process. Clusters are well mixed compared to before the correction process, which shows mitigated batch effects.

In this part of the experiment, we show the results of our pipeline on the integrated DLPFC dataset and the Coronal dataset. For the DLPFC dataset, we focused on utilizing the Case 1 integrated dataset of samples 151507, 151672, and 151673 as the benchmark dataset. We applied UMAP (McInnes *et al.* 2018) to visualize the existence of batch effects, revealing their evident presence within the UMAP plot. In Fig. 2, we show the visualization of the data distribution in a two-dimensional space, this visualization underscores the impact of batch effects on the underlying biological variability in the DLPFC dataset. We also show the UMAP plot of the Coronal dataset in Fig. 2, available as supplementary data at *Bioinformatics* online, which also reflects the similar conclusion that batches are distinctly separated.

By leveraging GSI, we conducted an examination of the UMAP plot, which revealed a pronounced batch effect within the dataset. In Fig. 2, the batch origins of the three integrated DLPFC samples were noticeably distinct, as well as the two batch origins of the Coronal dataset in Fig. 2, available as supplementary data at *Bioinformatics* online. After being processed using our GSI pipeline, these batch effects were systematically diminished, as illustrated by the before-and-after comparison in both figures. This outcome closely aligns with the ideal UMAP distribution, as depicted in Fig. 1d, available as supplementary data at *Bioinformatics* online, and was achieved while preserving the distinct separation of cell types and even enhancing its prominence. This underscores the effectiveness of our approach in uncovering biological signals previously masked by batch-induced variability.

In Fig. 1, available as supplementary data at *Bioinformatics* online, we also visualize our focus on the UMAP plot of the assay feature, which captured the inherent cellular homogeneity without spatial information. This plot is shown in Fig. 1a, available as supplementary data at *Bioinformatics* online, which serves as a baseline to gauge the extent of batch effects and spatial feature dynamics within the data.

However, evaluating only the assay feature is inadequate to draw conclusions. Our findings indicate that while the UMAP plot of assay features may not display distinct separation of batch clusters, incorporation of spatial information offers a contrasting perspective, as demonstrated in the distribution visualization presented in Fig. 1b, available as supplementary data at *Bioinformatics* online. Ignoring spatial information contained in spatial transcriptomics data can result in suboptimal analyses, potentially overlooking critical distinctions that influence gene expression within their spatial context. Therefore, we applied batch effect removal after incorporating spatial information to mitigate the distinct separation of batch clusters, as illustrated in Fig. 1c, available as supplementary data at *Bioinformatics* online. The final UMAP distribution demonstrates significant improvement.

To provide context for our approach, we present a comparison between our UMAP plot, shown in Fig. 1c, available as supplementary data at *Bioinformatics* online, and those generated by basic analysis without spatial information, shown in Fig. 1a, available as supplementary data at *Bioinformatics* online. This comparison highlights the superiority of GSI in capturing the nuances of gene expression patterns while preserving spatial context.

### 3.3 Quantitative assessment of GSI

To quantitatively assess our pipeline, we employed similar methodologies as described in (Fu *et al.* 2016), comparing the clustering results on the DLPFC dataset using the Adjusted Rand Index (ARI) score against other relevant methods. The ARI score evaluates the similarity between data clusters or partitions, with higher scores indicating better-clustered data. Therefore, a high ARI score suggests that more pronounced cell-type characteristics have been discovered. This measure requires ground truth to be established beforehand; therefore, we focused on the DLPFC dataset in this section. In a later section, we also provided a further evaluation following (Luecken *et al.* 2022) on measuring integration performance.

Our results indicate that GSI outperformed other methods, particularly current go-to methods for spatial transcriptomics analysis such as Seurat, Giotto (Dries *et al.* 2021), stLearn, and SpaGCN. Notably, GSI maintains a level of simplicity comparable to the base Seurat tools. This highlights our objective of adapting existing tools for assessing spatial datasets while retaining their operational familiarity.

Similarly to previous studies (Fu *et al.* 2016, Xu *et al.* 2024), we used brain slice 151673 of the DLPFC dataset as a benchmark to evaluate clustering performance using the ARI score. Compared to other methods, our performance score surpassed that of the base Seurat, Giotto, stLearn, and spaGCN. The results details are given in Table 1. While our GSI pipeline performs better than the major tools for ST data analysis, GSI performance alone may not be better than recent approaches based on graph neural networks such as GraphST (Long *et al.* 2023) and SEDR (Fu *et al.* 2016, Xu *et al.* 2024). However, since our approach maintains the Seurat object format as an output, with some adjustments, this offers more flexibility for other downstream analyses using existing tools, including the graph-based tools. We later demonstrate the advantage of this flexibility by combining our pipeline with a graph network-based pipeline as a greater joint pipeline and showcasing better analysis performance.

**Table 1.** Performance comparison between well-established methods for clustering analysis.^a^

Methods	ARI score
Seurat	0.225
stLearn (Pham *et al.* 2023)	0.308
Giotto (Dries *et al.* 2021)	0.367
SpaGCN (Hu *et al.* 2021)	0.383
GSI	0.405

a ARI score is measured on sample 151673 as a benchmark.

We also evaluated clustering results for brain slices 151507 and 151672, in addition to sample 151673, as we integrated these three datasets in our batch effect removal demonstration. Table 2 summarizes the impact of our treatment compared to using the base assay features of the ST dataset on clustering analysis using the Seurat tool. This includes a comparison with data processed using Harmony, a well-established tool in scRNA-seq batch effect mitigation. In Table 2, we refer to the dataset without spatial information as ‘Base’, the dataset with spatial information as ‘Spatial’, the dataset with batch effect removed using Harmony as ‘Harmony’, and the dataset with batch effect removed using our pipeline’s batch effect removal as ‘BEER’. Finally, our proposed pipeline method is noted as ‘Spatial + BEER’. The substantial increase in the ARI scores across all samples demonstrates how spatial embedding and data integration aid in uncovering the unique characteristics of each sample.

**Table 2.** ARI scores of the ablation study examining the impact of spatial information and batch effect removal.^a^

Case study	151507	151672	151673
Base	0.255	0.179	0.225
Base + Harmony	0.192	0.180	0.220
Base + BEER	0.219	0.191	0.225
Spatial	0.169	0.158	0.171
Spatial + Harmony	0.167	0.240	0.279
Spatial + BEER	**0.269**	**0.277**	**0.405**

^a^ Base = baseline assay only dataset, Spatial = spatial information augmented dataset, Harmony = batch effect removed dataset using Harmony, BEER = batch effect removed dataset using BEER. Bold numbers indicate the highest score for each sample.

**Figure 3.**
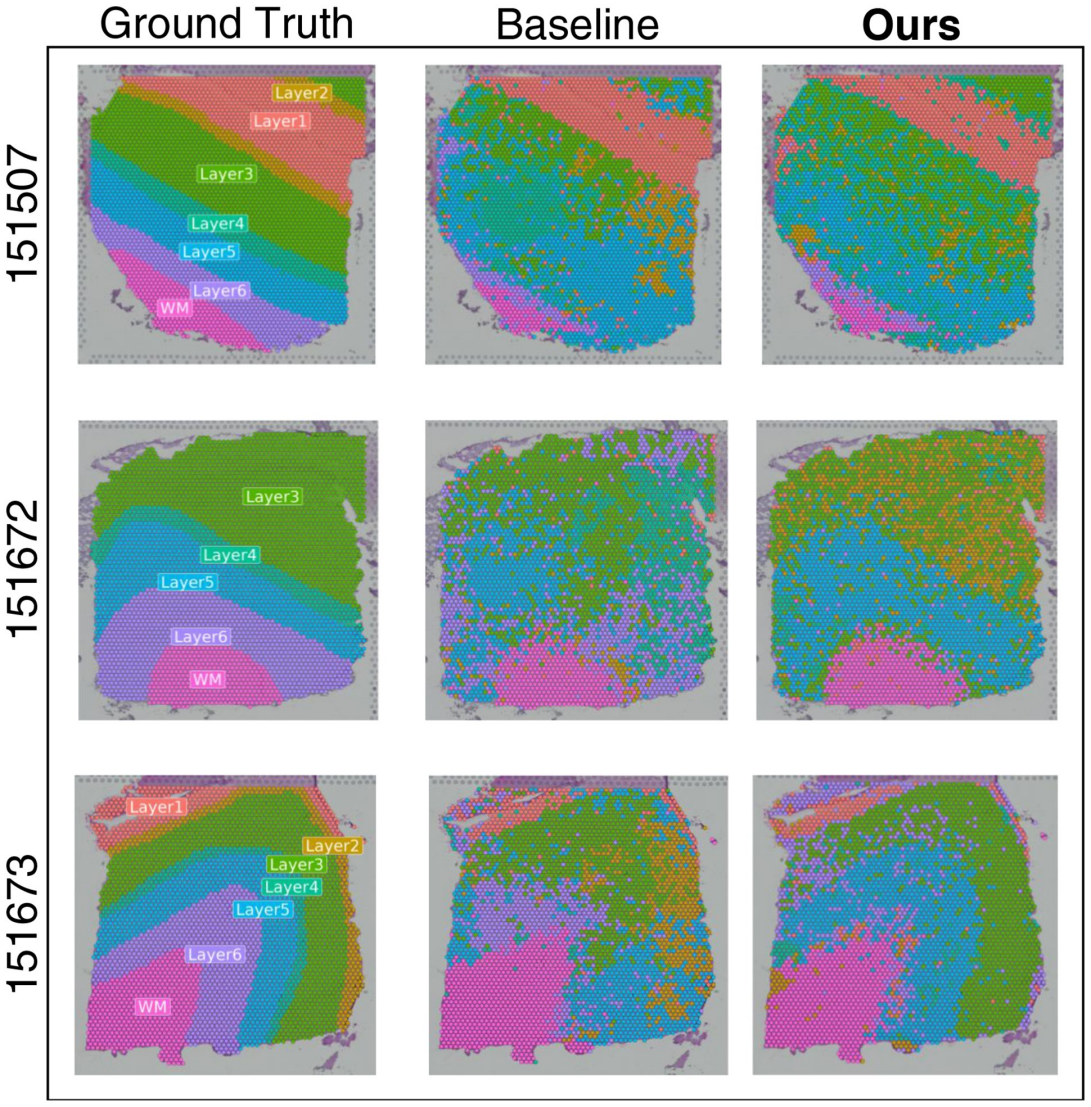
Performance visualization of Seurat tools in clustering tissue layers on the DLPFC spatial transcriptomics dataset without our treatment (Baseline) and after using our treatment (Ours) on the benchmark dataset. Each color represents a distinct cluster in relation to the original tissue layer.

In addition, we visualized how the clusters formed projected onto the actual tissue in Fig. 3 to better illustrate the improvement in specific aspects of the clustering evaluation, providing a more comprehensive understanding of our results. The differences are apparent, where the baseline method fails to identify a few prominent layers. After integrating spatial information with our pipeline, the layers are classified more distinctly over each other. Noticeable improvements are consistently observed when analyzing the integrated dataset using our pipeline compared to the untreated data.

To better show the impact of the increase in the ARI score, we visualized each cluster of layers in sample 151673 separately in Fig. 3, available as supplementary data at *Bioinformatics* online. As depicted in the figure, there is a notable enhancement in the clustering of Layer 3 and Layer 5 following our treatment. While some layers, such as WM and Layer 1, exhibited good clustering both visually before and after our treatment. Others, notably Layer 2, Layer 4, Layer 5, and Layer 6, proved difficult to identify. We conclude that this difficulty arises because there are no genes exclusively positioned to serve as markers for these layers, making our method less effective in identifying their spatial organization due to the lack of gene distribution patterns to learn from. The similarity measure for this comparison is provided in Fig. S4, available at *Bioinformatics* online.

We conducted further experiments to assess the impact of spatial embedding and integration of our pipeline through an ablation study. The results given in Table 2 highlight the impact of incorporating spatial feature embedding, integration, and removal of batch effects to improve the clustering performance of the Seurat analysis tool. When our method is applied, significant increases in the ARI score are observed, particularly in sample 151672 and in sample 151673, both nearly doubling the baseline Seurat performance. GSI added spatial context that helps the clustering algorithm identify layers with higher precision. This outcome underscores the success of our method in extracting important features from the underlying spatial information in spatial transcriptomics data and our integration approach across samples 151507, 151672, and 151673.

The resulting ARI scores demonstrate that without spatial information, the batch effect removal process leads to over-correction, which eliminates important cell-type features and decreases clustering performance. On the other hand, only adding spatial information without addressing batch effects significantly reduces the clustering performance, as noted previously, a prevalent batch effect is highly disruptive in the analysis process. Our approach in incorporating both spatial information and batch effect removal consistently improves clustering performance over the Base dataset, which only uses assay gene count information. In particular, the integration of our pipeline achieves the highest performance in all cases.

This study highlights how our pipeline uncovers more valuable information from the dataset by leveraging spatial information, data integration, and batch effect removal processes to enhance downstream analyses, such as clustering, compared to only using the base spatial transcriptomics gene expression features.

### 3.4 GSI as part of a greater pipeline

Our pipeline aims to improve spatial transcriptomics analysis by capturing unique spatial features that are not utilized by other existing methods. The extracted features are not exclusive and can be joined with other spatial features for a more thorough analysis. With this in mind, we further take advantage of this by integrating our pipeline with the state-of-the-art graph-based method, GraphST, in an additive manner. Using the joint pipeline (GraphST + GSI), we achieve a more thorough analysis using a graph neural network of GraphST on top of the integrated and batch-effect-removed output.

As described in Section 3.1, the DLPFC dataset was split into three separate groups to train neural networks, which are referred to as cases in this study. The aim was to determine whether various combinations of training data could focus more effectively on particular features of the tissue. We find that there is an increase in performance when we use the processed output of our pipeline compared to GraphST itself. In Case 1, we found an improvement in samples 151507 and 151672. Case 2 shows an overall increase in the ARI score over baseline, with notable changes in samples 151507, 151508, and 151509. Finally, Case 3 shows competitive performance results, although not as significant as previous cases, our pipeline shows an improvement in clustering sample 151670 and sample 151672. We summarize the details of these comparison results in Table 3.

**Table 3.** Performance comparison between GraphST and combined GraphST + GSI (Joint) pipeline in clustering benchmark dataset using ARI score.^a^

	Case 1			Case 2				Case 3			
Method	151507	151672	151673	151507	151508	151509	151510	151669	151670	151671	151672
GraphST	0.374	0.614	**0.636**	0.434	0.493	0.526	**0.489**	**0.591**	0.431	**0.613**	0.632
Joint	**0.533**	**0.795**	0.575	**0.535**	**0.520**	**0.566**	0.414	0.423	**0.457**	0.556	**0.652**

^a^ Bold values represent the highest-performing method for each sample.

To further evaluate the impact of our pipeline, we compared various biological conservation metrics and batch mixing metric scores, including Normalized Mutual Information (NMI), Average Silhouette Width (ASW), cell LISI score (clisi), isolated F1 (Iso. F1), isolated ASW (Iso. ASW), integration LISI score (ilisi), Batch ASW, graph connectivity (Graph Con.), and kBET. We evaluated pipeline performance in case 1 benchmark dataset under four conditions: using only baseline Seurat, only GSI, only GraphST, and GraphST combined with GSI. These results are calculated using the scib (Luecken *et al.* 2022) benchmarking library in Python. The complete results are presented in Table 2, available as supplementary data at *Bioinformatics* online.

When spatial information was introduced through our pipeline, all biological conservation metrics (ARI, NMI, ASW, clisi, iso. F1, and iso. ASW) show improvements. However, these improvements are followed by a slight decline in batch mixing scores (ilisi, batch ASW, Graph Con., and kBET). In conclusion, these results suggest that GSI enhances the biological relevance of the obtained embeddings, albeit with a modest increase in batch distinction. Nevertheless, the overall performance for cell characterization is better with GSI, outperforming both the Seurat baseline and graph-based GraphST results.

Lastly, we compared the results of our joint pipeline with other similar approaches, including Seurat, Giotto, SpaGCN, SpaceFlow, ConST, BayesSpace, STAGATE, and base GraphST. We selected sample 151672 as the benchmark for comparison. As the SOTA method, GraphST achieved the highest score of 0.63 among these approaches. Building on this, we supplemented GraphST with our pipeline, achieving an improved benchmark score of 0.79 in the ARI score, the highest in all methods. Detailed results are shown in Table 3, available as supplementary data at *Bioinformatics* online, results are cited from the GraphST study (Long *et al.* 2023), except Seurat and the joint pipeline.

The increase in performance demonstrates that our pipeline effectively learns critical features from both the integration process and the spatial distribution features, which were previously not utilized. A notable example is the performance increase in sample 151672 for benchmark Case 1, where incorporating our captured spatial features and integration raises the ARI score to 0.795, compared to the already strong 0.614 achieved by only using GraphST. Although performance improvements were not observed in all samples, the significant ARI improvements in many of the datasets indicate that important information is being uncovered through the inclusion of our pipeline. These results underscore the importance of spatial features of gene distribution, integration of the dataset, and removal of batch effects in the cell-type identification task.

## 4 Discussion and conclusion

Spatial transcriptomics serves as a powerful tool that links gene expression with the tissue context, providing multidimensional insights. The integration of spatial information enhances our understanding by unveiling contextual relationships between genes and cells within their native tissue environments. This technology remains a transformative field, as demonstrated by our innovative approach to processing gene distribution features as images, opening new avenues for analysis.

We introduce a novel method for transforming gene distribution into image instances, taking advantage of unexplored spatial aspects in spatial transcriptomics data. Leveraging techniques from image analysis, our approach effectively captures spatial information, broadens data representation, and enhances spatial feature extraction. By integrating our method into the analysis pipeline, the mitigated batch effects not only address data procurement limitations but also reveal biological signals otherwise obscured. The flexibility of combining with an existing pipeline adds advantages for our method to significantly improve analytical precision, advancing our understanding of spatial gene expression patterns and tissue-specific dynamics.

Notable improvements our pipeline achieves are the increase in performance over the baseline Seurat method across the benchmark set, evidence that there are important features discovered via our process. Followed by the improvement over the state-of-the-art method, GraphST, which, after being supplemented with our pipeline, achieves the highest ARI score on benchmark sample 151672 compared to other similar methods, this shows that the extracted features are also useful in the combined analysis process. These improvements show that our approach effectively takes advantage of gene distribution as spatial features, which were previously untapped in spatial transcriptomics analysis.

The integration of spatial transcriptomics data with image-based learning opens new paths for spatial analysis, fostering deeper insights. As spatial transcriptomics continues to evolve and more disease-related data become available, exploring the impact of spatial information on disease behavior will further shape our understanding of complex multidimensional biological processes.

## Supplementary Material

btaf350_Supplementary_Data

## Data Availability

DLPFC data are available at http://spatial.libd.org/spatialLIBD/. GSI implementation: https://github.com/Riandanis/Spatial_Integration_GSI and https://doi.org/10.5281/zenodo.15165223.
