# Supplementary material for: Gene spatial integration: enhancing spatial transcriptomics analysis via deep learning and batch effect mitigation
Source: Bioinformatics. 2025 Jun 13;41(6):btaf350. doi: 10.1093/bioinformatics/btaf350 (PMC12208067; doi:10.1093/bioinformatics/btaf350)
Supplement: btaf350_Supplementary_Data [file btaf350_supplementary_data.pdf]

1 Supplementary Notes

1.1 Auto Encoder training details

We use the autoencoder to perform representation learning on gene distribution data. This approach was chosen due to the autoencoder's ability to be trained in an unsupervised manner and its flexibility for future expansion (Meng *et al.*, 2017). Autoencoders are widely used for data reconstruction, making them a go-to method for unsupervised feature extraction (Zhang *et al.*, 2020). They are particularly effective in extracting spatial features from 2D maps (Egilmez and Ortega, 2014; Imani and Ghassemian, 2020; Cao *et al.*, 2021). Since we represent spatial information in spatial transcriptomics as map-like images, the autoencoder is an ideal fit for our approach.

In our pipeline, the autoencoder took the transformed gene distribution images P as input, learning to reconstruct P' to represent the spatial transcriptomic image data. The autoencoder consists of two parts: an encoder and a decoder. The encoder takes the image P as input and transforms it into a latent representation H. The decoder then takes the latent representation H as input and transforms it back into the image P'.

$$h_{enc_i}^{(0)} = \mathrm{ReLU}(W^{(0)} \cdot P_i + b^{(0)}) \tag{1}$$

$$h_{enc_i}^{(l)} = \mathrm{ReLU}(W^{(l)} \cdot h_{enc_i}^{(l-1)} + b^{(l)}), \quad \text{for } l = 1, 2, \ldots, L-1 \tag{2}$$

$$H_i = \mathrm{ReLU}(W^{(L)} \cdot h_{enc_i}^{(L-1)} + b^{(L)}) \tag{3}$$

where $h_{enc_i}^{(l)}$ is the hidden representation output of the encoder layer l^{th} of the gene distribution image P for gene i, the encoder uses the Rectified Linear Unit $ReLU$ as the activation function, and the weight matrix W as a parameter and also the bias vector b. Our architecture consists of $L = 4$ layers in total, each layer of the encoder is a fully connected feed forward neural network, condensing the input of 2D image to lower dimension feature embedding. We chose a basic feed-forward neural network for the encoder layer because this approach extracts features from the entire distribution as a singular interconnected instance. This allows us to investigate whether useful spatial information is contained within. The final layer outputs H_i, which is then used as input to the decoder to reconstruct the image P as P'.

$$h_{dec_i}^{(0)} = \mathrm{ReLU}(W^{(0)} \cdot H_i + b^{(0)}) \tag{4}$$

$$h_{dec_i}^{(l)} = \mathrm{ReLU}(W^{(l)} \cdot h_{dec_i}^{(l-1)} + b^{(l)}), \quad \text{for } l = 1, 2, \ldots, L-1 \tag{5}$$

$$P'_i = \mathrm{ReLU}(W^{(L)} \cdot h_{dec_i}^{(L-1)} + b^{(L)}) \tag{6}$$

where $h_{dec_i}^{(0)}$ the decoder initial layer output, then following $h_{dec_i}^{(l)}$ the output of consequent decoder layers. With P'_i as the spatial distribution reconstruction of gene i. Similar to the encoder, we use the Rectified Linear Unit $ReLU$ as the activation function, with a weight matrix W as the parameter and a bias vector b. The decoder module consists of $L = 4$ layers, matching the number of layers in the encoder for better reconstruction of the encoded features. In this way, the hidden representation H captures the spatial information from images P.

$$\mathcal{L}_{recon} = \frac{1}{N_{gene}} \sum_{i=1}^{N_{gene}} \|(P_i, {P'}_i)\|_2 \tag{7}$$

By minimizing the mean square error loss function $\mathcal{L}_{recon}$, the autoencoder network is trained to adjust the parameters W, b_{P_i}, and b_{H_i} to learn a representation H that can reconstruct the image P' as closely as possible to P. The hidden feature H a reduced representation of the spatial information from the image P, which proves useful for various analysis tasks, including cell clustering, cell type identification, and gene differential expression analysis.

In our experiment, we used an autoencoder network implemented in PyTorch (Imambi *et al.*, 2021) with the following architecture:

1. Encoder with an input layer that takes (59400, 1) features, four hidden layers, and an output layer that gives (N_{spot}, 1) features.
2. Decoder with one input layer taking (N_{spot}, 1) features, four hidden layers, and an output layer giving (59400, 1) features.

The neural network was trained with a 70-30 train-test split for 100 epochs and a learning rate of $1e-3$ with Adam optimization. Early stopping was also implemented to prevent overfitting.

1.2 Batch integration evaluation metrics

The evaluation metrics to measure pipeline performance in data integration were performed using biological metrics proposed by (Luecken *et al.*, 2022). These metrics are categorized into two categories of metrics, which are: (1) Measure of Batch mixing and (2) Conservation of biological signal. The batch mixing measure includes batch ASW, graph connectivity, iLISI, and kBET. The second category, conservation of biological signal, includes ARI, NMI, cell ASW, cLISI, isolated F1, and isolated ASW. The evaluation is implemented in a Python environment using the scib library package, using the embedding output of the measured pipeline as input.

1.2.1 Adjusted Rand Index (ARI) score clustering metric

The clustering evaluation metric used is the Adjusted Rand Index (ARI) (Chacón and Rastrojo, 2023), which measures the similarity between two data clusters specified by a random model. This metric is chosen for evaluation due to the unsupervised nature of the analysis, where the number of clusters resulting from the clustering process is not specified. ARI yields a value between -1 and +1, with higher values indicating better clustering performance.

Given a set S of n elements and two clusters of these elements, namely $X = \{X_1, X_2, ..., X_r\}$ and $Y = \{Y_1, Y_2, ..., Y_s\}$, with the overlap between X and Y summarized in a $r \times s$ contingency table $[n_{ij}]$ where each entry n_{ij} denotes the number of objects in common between X_i and Y_j such that $n_{ij} = |X_i \cap Y_j|$. The ARI score of such a set is calculated as follows:

$$ARI = \frac{\Sigma_{ij}\binom{n_{ij}}{2} - [\Sigma_i\binom{a_i}{2}\Sigma_j\binom{b_j}{2}]/\binom{n}{2}}{\frac{1}{2}[\Sigma_i\binom{a_i}{2} + \Sigma_j\binom{b_j}{2}] - [\Sigma_i\binom{a_i}{2}\Sigma_j\binom{b_j}{2}]/\binom{n}{2}} \tag{8}$$

Where n_{ij}, a_i, b_j are the values from the contingency table with $a_i = \Sigma_{j=1}^{r} n_{ij}$ the total sum in rows of X_i and $b_j = \Sigma_{i=1}^{s} n_{ij}$ the total sum in columns of Y_j.

The yielded value is then scaled to scores of 0 to 1, where 1 corresponds to a perfect match.

1.2.2 Normalized Mutual Information (NMI) score clustering metric

NMI quantifies the agreement between the clustering results and known cell-type labels, serving as a measure of biological signal preservation. NMI evaluates the shared information between two label assignments with a normalized score in the range of 0 to 1, where 1 indicates perfect agreement and 0 denotes no mutual information.

1.2.3 Average Silhouette Width (ASW) score

Silhouette width measures the distance between the cell and other cells within the same label group versus the nearest neighboring group (Rousseeuw, 1987). ASW scores range from -1 to 1, where 1 indicates better cohesion within clusters and well separation between clusters, while scores from -1 to 0 indicate overlapping clusters and misclassifications. In our implementation, we make use of ASW scores to measure cell type clustering as ASW score, and to measure batch mixing as Batch ASW score. To obtain ASW score we use the following formula:

$$ASW = (ASW_C + 1)/2 \tag{9}$$

With C representing the possible cell types.

For obtaining Batch ASW score we use the following formula:

$$batchASW = \frac{1}{M}\Sigma_{i \in C_j} 1 - s_{batch}(i) \tag{10}$$

Where C_j is the set of cells with cell type label j and $s_{batch}(i)$ is the silhouette width on the batch labels per cell i. The resulting ASW score and the Batch ASW score are scaled to have values between 0 and 1. Higher scores indicate better clustering or batch mixing.

1.2.4 Graph LISI scores

Local Inverse Simpson Index (LISI) metrics implemented in (Korsunsky *et al.*, 2019). LISI measures both biological conservation and batch mixing after data integration by quantifying local label diversity within the neighborhood of each cell in an embedding space. Two variants were used: Cell-type LISI (cLISI) and integration LISI (iLISI). cLISI measures biological separability by computing cell-type label annotations, where lower values (approaching 1) indicate that neighboring cells mostly share the same cell type, reflecting a well-preserved biological structure. iLISI, on the other hand, evaluates batch mixing by computing label diversity across batch label annotations, with higher iLISI values indicating that local neighborhoods are composed of cells from multiple batches, indicating effective integration. LISI scores range from 1 to N, where N is the number of batches, indicating perfect separation and perfect mixing.

With B denoting the total number of batches, we used the following formula for scaling cLISI score:

$$cLISI : f(x) = \frac{B - x}{B - 1} \tag{11}$$

As for iLISI, we use the following formula to scale the score:

$$iLISI : g(x) = \frac{x - 1}{B - 1} \tag{12}$$

The resulting score ranges from 0 to 1, with a higher value indicating better cell type separation and better batch integration.

1.2.5 Isolated label scores

SCIB benchmark tools (Luecken *et al.*, 2022) determine the isolated cell labels as the labels present in the least number of batches in the integration. Following such an example, we use two versions of the isolated label metric: (1) measure the best clustering of the isolated label using the F1 score and (2) measure the global ASW of the isolated label. The F1 score is a weighted mean of precision and recall, calculated using the following formula:

$$F_1 = 2 \times \frac{precision \times recall}{precision + recall} \tag{13}$$

The resulting score is in the range of 0 to 1, where 1 shows that the isolated label cells and no other labels are present in the cluster. For the isolated ASW score, we calculate the ASW score of the isolated labels versus the nonisolated labels. The resulting score also scaled to be between 0 and 1.

1.2.6 Graph connectivity

Graph connectivity evaluates the extent to which cells sharing the same identity form connected components within the integrated graph of k-nearest neighbor (kNN). The graph connectivity score (GC) is computed using the formula below:

$$GC = \frac{1}{|C|} \Sigma_{c \in C} \frac{|LCC(G(N_c; E_c))|}{|N_c|} \tag{14}$$

where C represents the set of cell-type labels, $|LCC()|$ refers to the size of the largest connected component of the graph and $|N_c|$ is the number of nodes annotated with label c. The score ranges from 0 to 1, with a value of 1 indicating that all cells of the same type are fully connected on the kNN graph. A lower score indicates poor connectivity, with 0 corresponding to a graph in which no cells of the same type are connected.

1.2.7 k-Nearest Neighbor Batch Effect Test (kBET)

kBET was used to quantitatively assess batch mixing after integration. KBET evaluates whether the local composition of batch labels in the neighborhood of each cell deviates significantly from the global batch distribution (Büttner *et al.*, 2019). The test is repeated for a random subset of cells, resulting in a rejection rate in the tested neighborhood. The resulting score ranged from 0 to 1, where 1 denotes poor batch removal. To be consistent with the scoring scale, the score is subtracted from 1 so that the higher final score reflects better performance.

1.2.8 Metric aggregation

Overall metric is also added by calculating the weighted average of all the results score following this formula:

$$\text{Overall Score} = 0.6 \cdot \left(s_i^{\text{bio}}\right) + 0.4 \cdot \left(s_j^{\text{batch}}\right) \tag{15}$$

where s^{bio} is the average of the biological signal conservation metric score, and s^{batch} is the average of the batch mixing metric score.

Supplementary Figures

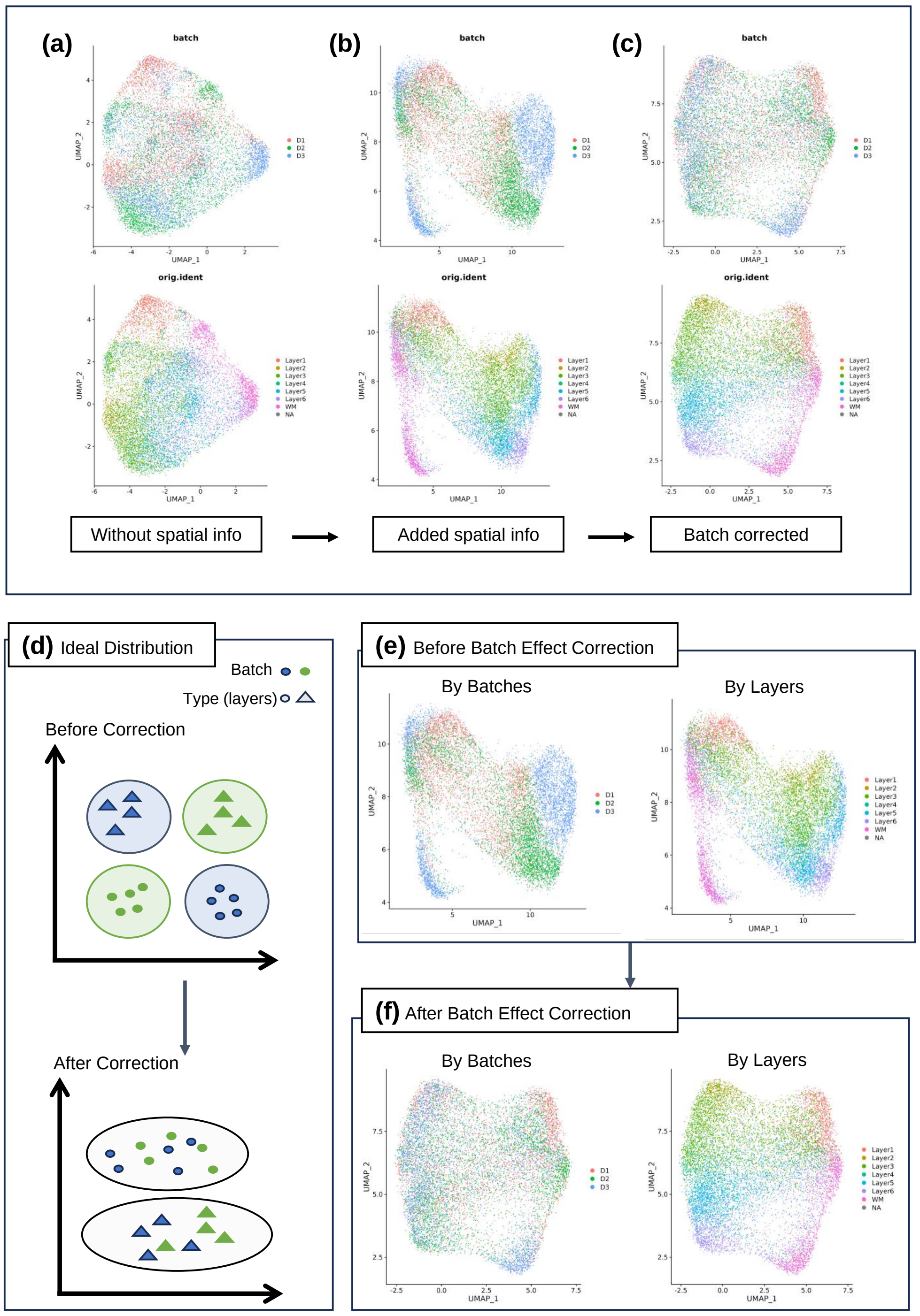

Fig. S1. Visualization of the evolving distribution throughout our pipeline treatment: (a) from base assay features with layer clusters not visibly separated despite batch clusters are close to ideal, (b) to incorporation of spatial information embedding that emphasize layer clusters but amplifies batch effect, and (c) after batch effect removal treatment that maintains layer clusters distinction while mitigating batch effect. (d) Illustration of the ideal distribution before and after batch effect removal, with batch labels in separate clusters while maintaining similar layer labels within the same cluster. (e) UMAP Visualization demonstrating the presence of batch effects before our pipeline treatment on the integrated DLPFC dataset. (f) Resulting UMAP visualization after the batch effect removal process of our treatment on the integrated DLPFC dataset.

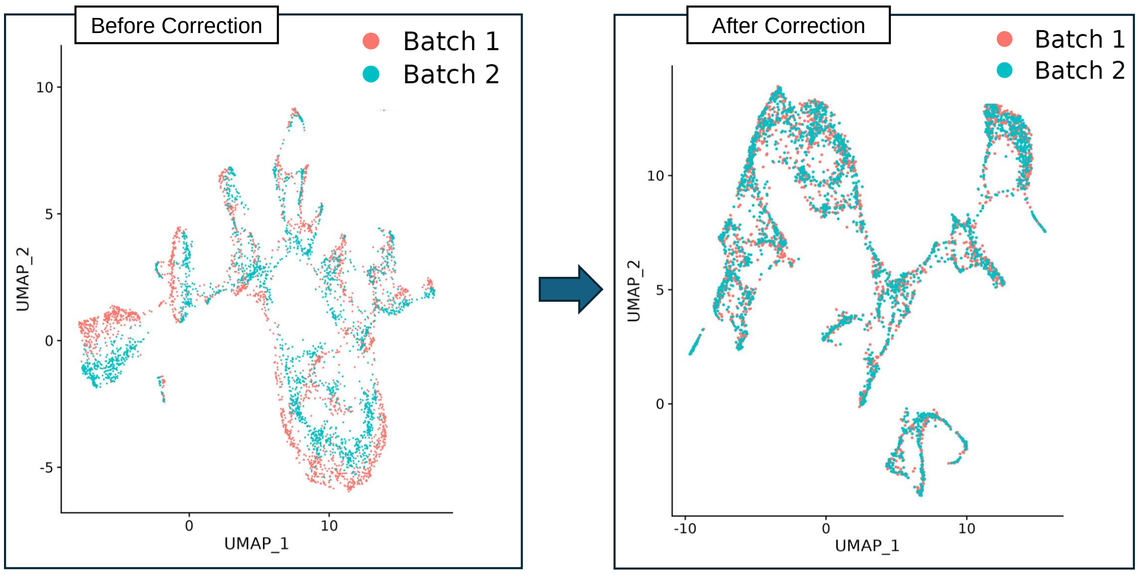

Fig. S2. Visualization of batch clusters before and after batch effect removal process of Mouse Brain Coronal dataset. Clusters are well mixed compared to before correction process which shows mitigated batch effects

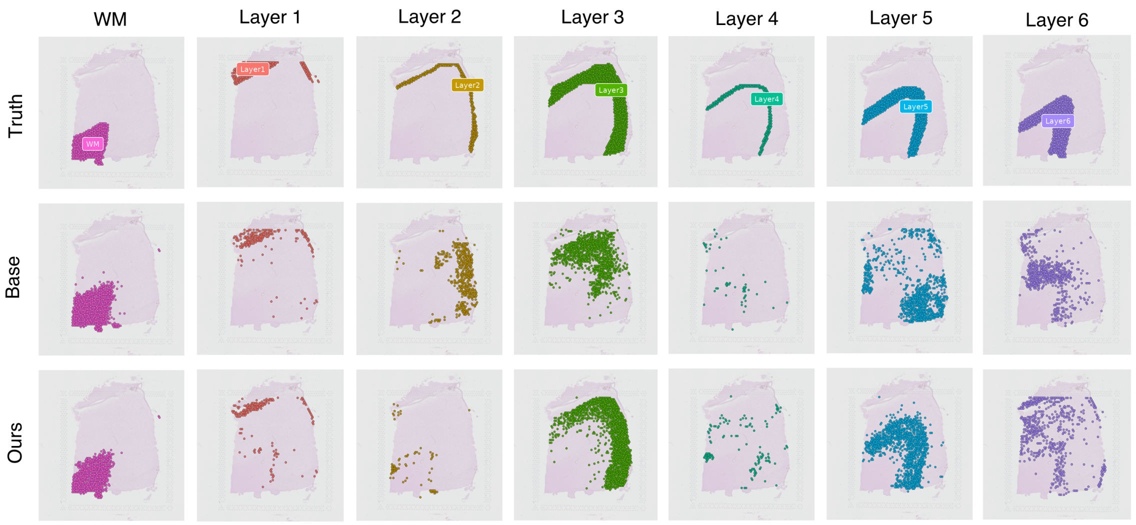

Fig. S3. Visualization of per-layer cluster results of sample 151673 comparing the dataset without our treatment (Base) to the dataset after our treatment (Ours). While both WM and Layer 1 exhibit adequate clustering, notable improvements are observed in Layer 3 and Layer 5.

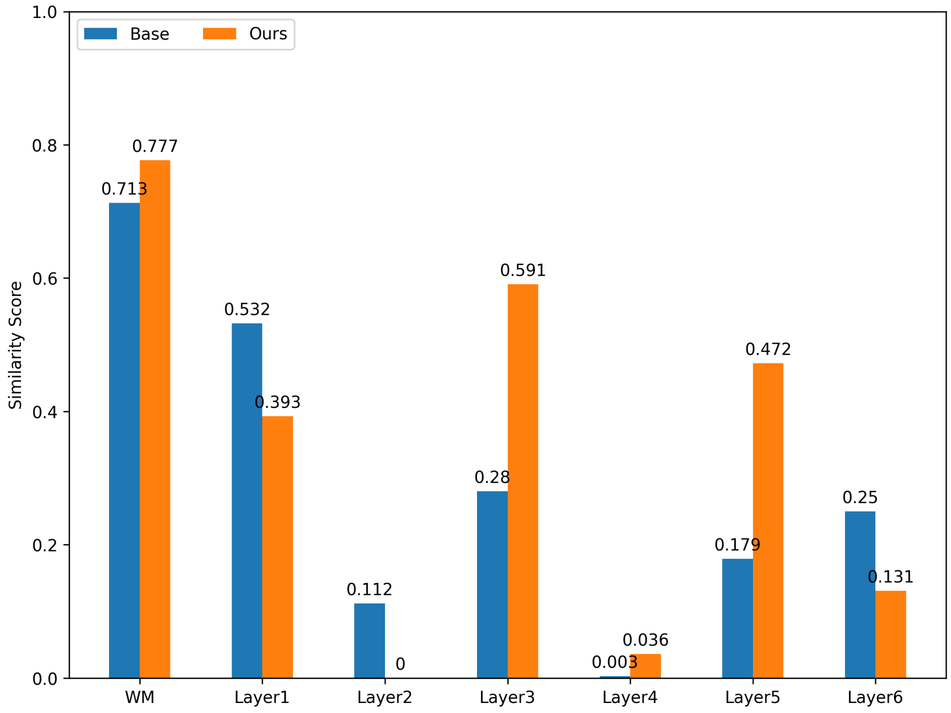

Fig. S4. Summary of Jaccard similarity metrics for each layer of sample 151673 comparing the results obtained using GSI against the base dataset.

Supplementary Tables

Table S1. Summary of dataset grouping selection as separate Cases.

Group	Subject tissue	Dataset samples
Case 1	Br5292, Br5595, Br8100	151507, 151672, 151673
Case 2	Br5292	151507, 151508, 151509, 151510
Case 3	Br5595	151669, 151670, 151671, 151672

Table S2. Metric scores calculated using scib benchmarking tool of the pipeline variations examining the impact of spatial information and batch effect removal (scaled to 0 to 1, where 1 indicates the best performance).

Method	Biological Conservation										Batch Mixing				Overall
	ARI			NMI			ASW	clisi	Iso. F1	Iso. ASW	ilisi	Batch ASW	Graph Con.	kBET	
	151507	151672	151673	151507	151672	151673									
Base + BEER	0.219	0.183	0.222	0.318	0.241	0.323	0.488	0.758	0.442	0.484	**0.733**	**0.933**	0.855	**0.230**	0.496
GSI	0.249	0.260	0.384	0.347	0.333	0.435	**0.495**	0.807	**0.563**	**0.531**	0.655	0.924	0.866	0.212	0.530
GraphST	0.374	0.614	**0.636**	0.584	0.718	**0.735**	0.499	**0.863**	0.555	0.530	0.431	0.859	**0.994**	0.02	0.597
Joint	**0.534**	**0.795**	0.575	**0.668**	**0.731**	0.698	0.496	0.784	0.529	0.5	0.544	0.863	0.892	0.016	**0.613**

Table S3. ARI scores comparison in clustering benchmark sample 151672, examining performance of existing similar methods in comparison to the proposed joint pipeline.

**Methods**	**ARI score**
Seurat	0.18
Giotto (Dries *et al.*, 2021)	0.45
SpaGCN (Hu *et al.*, 2021)	0.57
SpaceFlow (Ren *et al.*, 2022)	0.51
conST (Zong *et al.*, 2022)	0.62
BayesSpace (Zhao *et al.*, 2021)	0.43
STAGATE (Dong and Zhang, 2022)	0.59
GraphST (Long *et al.*, 2023)	0.63
**GraphST + GSI**	**0.79**

References

Büttner, M. *et al.* (2019). A test metric for assessing single-cell rna-seq batch correction. *Nature methods*, **16**(1), 43–49.

Cao, Z. *et al.* (2021). Contrastnet: Unsupervised feature learning by autoencoder and prototypical contrastive learning for hyperspectral imagery classification. *Neurocomputing*, **460**, 71–83.

Chacón, J. E. and Rastrojo, A. I. (2023). Minimum adjusted rand index for two clusterings of a given size. *Advances in Data Analysis and Classification*, **17**(1), 125–133.

Dong, K. and Zhang, S. (2022). Deciphering spatial domains from spatially resolved transcriptomics with an adaptive graph attention auto-encoder. *Nature communications*, **13**(1), 1739.

Dries, R. *et al.* (2021). Giotto: a toolbox for integrative analysis and visualization of spatial expression data. *Genome biology*, **22**, 1–31.

Egilmez, H. E. and Ortega, A. (2014). Spectral anomaly detection using graph-based filtering for wireless sensor networks. In *IEEE International Conference on Acoustics, Speech and Signal Processing (ICASSP), 2014*, pages 1085–1089. IEEE.

Hu, J. *et al.* (2021). Spagcn: Integrating gene expression, spatial location and histology to identify spatial domains and spatially variable genes by graph convolutional network. *Nature methods*, **18**(11), 1342–1351.

Imambi, S. *et al.* (2021). Pytorch. *Programming with TensorFlow: Solution for Edge Computing Applications*, pages 87–104.

Imani, M. and Ghassemian, H. (2020). An overview on spectral and spatial information fusion for hyperspectral image classification: Current trends and challenges. *Information fusion*, **59**, 59–83.

Korsunsky, I. *et al.* (2019). Fast, sensitive and accurate integration of single-cell data with harmony. *Nature methods*, **16**(12), 1289–1296.

Long, Y. *et al.* (2023). Spatially informed clustering, integration, and deconvolution of spatial transcriptomics with graphst. *Nature Communications*, **14**(1), 1155.

Luecken, M. D. *et al.* (2022). Benchmarking atlas-level data integration in single-cell genomics. *Nature methods*, **19**(1), 41–50.

Meng, Q. *et al.* (2017). Relational autoencoder for feature extraction. In *2017 International joint conference on neural networks (IJCNN)*, pages 364–371. IEEE.

Ren, H. *et al.* (2022). Identifying multicellular spatiotemporal organization of cells with spaceflow. *Nature communications*, **13**(1), 4076.

Rousseeuw, P. J. (1987). Silhouettes: a graphical aid to the interpretation and validation of cluster analysis. *Journal of computational and applied mathematics*, **20**, 53–65.

Zhang, G. *et al.* (2020). A survey of autoencoder-based recommender systems. *Frontiers of Computer Science*, **14**, 430–450.

Zhao, E. *et al.* (2021). Spatial transcriptomics at subspot resolution with bayesspace. *Nature biotechnology*, **39**(11), 1375–1384.

Zong, Y. *et al.* (2022). const: an interpretable multi-modal contrastive learning framework for spatial transcriptomics. *BioRxiv*, pages 2022–01.

[h!]
